# Testing Positive and Disclosing in Pregnancy: A Phenomenological Study of the Experiences of Adolescents and Young Women in Maseru, Lesotho

**DOI:** 10.1155/2020/6126210

**Published:** 2020-02-12

**Authors:** Sphiwe Madiba, Mamorapeli Putsoane

**Affiliations:** Department of Public Health, Sefako Makgatho Health Sciences University, Pretoria, South Africa

## Abstract

The routine antenatal screening through the prevention of mother to child transmission of HIV (PMTCT) services results in pregnancy being often the point at which an HIV diagnosis is made. Disclosure to partners presents particular complexities during pregnancy. However, research on the pattern and experiences of disclosure in pregnancy is limited in Lesotho, despite the high prevalence of HIV among pregnant women. The aim of this study was to explore and describe the disclosure experiences of adolescent girls and young women (AGYW) after receiving a positive HIV test result during pregnancy. *Methods*. Descriptive phenomenology using semistructured in-depth interview was used to collect data from AGYM sampled purposively from PMTCT sites located in urban areas of Maseru, Lesotho. Data analysis was inductive and followed the thematic approach. *Findings*. There were 15 AGYW involved in this study with the mean age of 20 years. Fourteen reported being pregnant with their first child and perceived HIV testing in antenatal care as compulsory. Ten AGYM disclosed their HIV status in the immediate posttesting period to protect their partners from HIV infection. The narratives revealed that the AGYM hoped that after disclosing, the partner would be tested for HIV. Furthermore, the AGYM disclosed because they wanted freedom to take their medication. Their experience of disclosure was relief, as they did not have to hide their HIV status. The AGYM reported being supported to adhere to medication and clinic attendance by their partners who also provided emotional support to them to deal with being HIV positive and pregnant. *Conclusion*. The AGYM recounted an overall positive experience of disclosure to their partners who agreed to test for HIV and adopted safe sex practices. This has positive implications for the PMTCT programme and the involvement of men in reproductive health. Therefore, there is need to integrate disclosure and partner testing interventions in the cascade of services in PMTCT programmes.

## 1. Introduction

In sub-Saharan Africa (SSA), adolescent girls and young women (AGYM) account for a 25% of new HIV infections among adults, despite interventions to reduce the rates of new infections [1]. Globally, young women aged 15 to 24 are twice as likely to be infected with HIV as their male counterparts [2]. Lesotho is one of the countries in SSA that is heavily affected by the HIV epidemic with general prevalence of HIV estimated at 25.6% [3].

Data suggest an increasing rate of acquiring HIV during pregnancy among adolescent girls and young women (AGYM) [4–6]. As a result of routine antenatal screening through the prevention of mother to child transmission of HIV (PMTCT) services, pregnancy is often the point at which an HIV diagnosis is made [7, 8]. The adoption of the provider-initiated testing and counselling (PITC) as an opt-out approach to HIV testing and counselling (HTC) in the PMTCT programme has increased uptake of HIV testing services and early diagnosis of HIV among pregnant women [3]. This has led to a high proportion of pregnant adolescents in many settings in SSA learning about their HIV-positive status for the first time during antenatal screening [8–11].

A critical component of the PMTCT services is antenatal care services which play a critical role of linking women to HIV testing, pregnancy screening and monitoring, initiation and use of antiretroviral treatment (ART), and postnatal care of the mother and her infant [8, 12]. However, the success of the PMTCT services depends on the ability to retain the women in care during the prenatal and postnatal period. HIV status disclosure, particularly to partners, remains central to increasing women's participation in PMTCT programmes [12–14]. Disclosure is particularly important within the Option B+ guidelines for PMTCT, whereby HIV-positive pregnant women are initiated on lifelong ART regardless of CD4 count [15, 16]. Lack of disclosure and partner support impacts negatively on the prevention of MTCT of HIV, increases the risk of disengagement from care, results in poor adherence to recommended ART, decreases facility childbirth, and increases poor adherence to a breast feeding regimen [12, 17, 18].

The risk for the suboptimal uptake of PMTCT services remains high in SSA because of nondisclosure during pregnancy. A systematic review of disclosure studies in SSA found low disclosure rates among pregnant and postpartum women compared with the general population of people living with HIV (PLHIV) [19]. Whereas disclosure is challenging and complex at any period for PLHIV; it presents particular complexities during the vulnerable period of pregnancy and the postpartum period [12]. Receiving HIV-positive results during pregnancy is a distressing experience for women. The situation of having to deal with pregnancy, fear of transmission of HIV to the baby, disclosure of HIV status, and the decision to start lifelong ART make them vulnerable to multiple emotions [7]. Therefore, HIV disclosure, particularly to partners, remains a significant source of stress because women often face the dual burden of disclosing both their HIV status and their pregnancies [20].

Low rates of disclosure that are observed among women who test HIV positive during pregnancy are due to fear of anticipated negative reactions, especially from partners [18]. The reasons cited for nondisclosure to partners during pregnancy include fears and concerns of losing support from their partners, of being accused of infecting their partner, of abandonment or rejection, violence from their partners, fear of accusation of infidelity, and fear of the loss of emotional and financial support for both the mother and the child [13, 14, 20, 21].

HIV disclosure to male partners is a significant source of stress for pregnant women [18, 20]. However, research on the pattern and experiences of disclosure to partners during pregnancy is limited in Lesotho, despite the high prevalence of HIV among pregnant women. The Lesotho ANC Surveillance (2016) estimates the HIV prevalence among pregnant women to be 27.9%, and AGYW are disproportionally affected. In general, research on the unique challenges of disclosure for pregnant women in SSA is limited as the few available studies were conducted in developed countries [9, 15]. The aim of this study was to explore and describe the disclosure experiences of AGYW after receiving a positive HIV test result during pregnancy. The PMTCT programmes have a key role to play in helping pregnant women to make decisions about HIV disclosure and assisting them to navigate the disclosure process with partners [18, 20].

## 2. Materials and Methods

### 2.1. Study Design and Setting

The study settings were PMTCT sites located in urban areas of Maseru, the capital of the Kingdom of Lesotho. The Kingdom of Lesotho is a small (30,000 square kilometres in space) landlocked country surrounded by South Africa. Lesotho has an estimated population of 2.24 million; about 34% of the population live in urban and 66% in rural areas. Maseru lies directly on the Lesotho-South Africa border and has a total population of 4,74,791. Women of childbearing age are estimated to be 1,43,676 [22]. The HIV prevalence in Lesotho in 2015 was estimated to be 22.7%, and about half of women under 40 years are living with HIV [3]. In Maseru where the study was conducted, there are about 60 public and private health facilities providing PMTCT services and other HIV related services. All the facilities provide PMTCT and other HIV services using national HIV treatment guidelines. Three facilities were selected for the study, two were public clinics and one was private owned. The National Health Surveillance system estimates that the three PMTCT study sites see an average of 400 pregnant AGYW per month [22].

Descriptive phenomenology was used to explore and gain an in-depth understanding of how AGYW experience disclosure to their partners after testing HIV positive during their pregnancy. Descriptive phenomenology is a method of choice to understand and describe a phenomenon as experienced by a group of people without attempting to predict or explain behaviour [23, 24]. Consistent with phenomenological enquiry, the researcher used purposive sampling to select AGYW who have experienced an HIV diagnosis during pregnancy [25] and to explore their perceptions, perspectives, understandings, and feelings about the phenomenon. In purposeful sampling, the researcher selects participants who can offer a meaningful perspective on the phenomenon of interest, from whom the researcher can learn a great deal about the phenomenon under inquiry [26]. The sample size consisted of 15 AGYW because the goal of phenomenological research is not to create results that can be generalised, but to understand the meaning of an experience of a phenomenon. Therefore, phenomenology emphasises rich qualitative accounts over the quantity of data. As a result, a small number of homogenous participants is studied through extensive and prolonged engagement to develop patterns and relationships of meaning [27]. In the current study, homogeneity was maintained by selecting only participants who were HIV positive and pregnant. However, variability and diversity in terms of sociodemographics such as age, marital status, educational status, and parity ensured variation in the information to reduce information bias [26].

### 2.2. Data Collection

Semistructured interview guide was used to collect data from the participants using a self-developed interview schedule with open-ended questions and possible probes. Consistent with the tradition of phenomenology [24, 28, 29], the participants were asked two broad, general questions: (1) what was it like to disclose your HIV positive status to your partner? (2) What was the outcome of disclosing your HIV positive status to partner? These questions were used to gather data to come to a deeper understanding of the nature or meaning of their experiences of testing positive during pregnancy and disclosing their HIV status to partners. The investigator (second author) asked other questions and followed up on points raised in responses to the given questions. In addition, the interview schedule was used flexibly to allow the participants to speak freely about their experience of the phenomenon in their own words [24, 28]. To allow the participants to speak freely, the interviews were conducted in Sesotho, the local language. The participants were interviewed once, and the interviews were 30–45 minutes long and were recorded with consent.

### 2.3. Data Analysis

To prepare for data analysis, the investigator and a trained research assistant transcribed verbatim the interviews, translated them into English, and checked the transcripts for accuracy against the audio recordings. Data analysis was inductive and followed the thematic approach grounded in the tradition of descriptive phenomenology [30] as outlined in Sundler et al. [30]. The recordings were listened to and transcripts read several times to get an overall sense of the data and develop familiarity with the phenomenon that was being described. Next, the investigators searched and extracted statements for meanings to uncover emergent themes that described the live experiences of the participants. Lastly, the emergent themes were integrated and synthesized into a meaningful one that captured the phenomenon as experienced by the participants. The NVivo (QSR International, Melbourne, Australia), a qualitative data analysis package, was utilised for the analysis process.

In phenomenology, rigour is ensured through the thoroughness and completeness of the data collection and analysis [31]. To engender rigour, the researcher adopted a reflective attitude throughout the data analysis to ensure that interpretation was free of bias [31], and credible conclusions were being drawn from the data and that procedures were being followed to ensure a quality study.

### 2.4. Ethical Considerations

Ethical clearance for this study was obtained from the Research and Ethics Committee of Sefako Makgatho Health Sciences University (SMUREC/H/121/2017: PG) and Lesotho National Ethics Committee. The participants were informed that their participation was voluntary. All provided written informed consent before the interviews. Pseudonyms were used to present the quotations to ensure privacy and anonymity.

## 3. Findings

### 3.1. Participant Context

Table 1 provides participants' demographic characteristics. There were 15 participants involved in this study, and their ages ranged from 18 to 24 years of age with the mean age of 20 years. None was employed nor did schooling during the study period. Educational levels varied from primary education (3), secondary education (10), to tertiary education (2). Marital status varied, with ten reporting that they were married and five reported being single. The married AGYW reported that they disclosed to their partners while the five single ones did not disclose because they had permanent partner separation before they could disclose their HIV test results. All the AGYW reported that they disclosed to family members (parents, grandparents, and siblings) while none extended the disclosure to the people outside of their families. Five of those who were married reported that their partners tested negative, and the five women who were abandoned did not know the HIV status of their partners. Fourteen AGYW reported being pregnant with their first child.

### 3.2. Emergent Themes

Seven themes emerged from the analysis of the interviews, which helped to capture the participants' experiences of disclosure during pregnancy. The main themes include the following: (1) HIV testing is not optional; (2) disclosure is a personal choice; (3) knowing the partner's status facilitates disclosure; (4) the need to protect the partner; (5) feeling relieved; (6) disclosure facilitates partner testing; and (7) feeling supported by the partner.

#### 3.2.1. HIV Testing Is Not Optional

HIV counselling and testing are offered to all pregnant women who enroll in the PMTCT programme. It is the first step in the PMTCT programme and the entry point for women to receive other PMTCT services. Thirteen of the 15 AGYW in the study discovered their HIV-positive status during their first antenatal visit. Their narratives indicated that when they visited the clinics they did not know that antenatal screening included HIV testing.

I think I never had any interest about HIV, I was just living my life recklessly, I never cared to get tested, I never thought of it, I never felt the need to get tested, I don't know why, I think I was just being ignorant, and I had not even a slight idea about HIV. I heard them mention that HIV testing will be done and I never thought I also had to get tested because I was not feeling sick. I thought I could only get tested when I am sick, but that was caused by being clueless or not being well informed. (Nthateng, 18 years old)

When we were at the clinic, we were told to go for the HIV test. I would not have been tested for HIV if I were not pregnant. We were told to get tested for HIV in the counselling room. (Dudu, 20 years old)

The AGYW perceived the HIV testing as an activity that was expected of them because of the pregnancy but also indicated that they felt that they had no choice but to do it.

It was a matter of must to test because I was pregnant. We were told that we were supposed to get tested and know our statuses so that if ever we test positive we must take the treatment to save the baby's' live so that the baby will be born HIV free. (Nthateng, 18 years old)

We were told that everyone has to be tested; no one should leave without being tested first. They further told us that when we come for the test you should bring your partner if you have one. After testing HIV positive one should start taking ART so that the baby is born free of HIV. (Thandy, 24 years old)

#### 3.2.2. Disclosure Is a Personal Choice

Ten women had disclosed their HIV-positive test results to the father of the baby soon after testing. Those who were abandoned by the father of the baby after being informed about the pregnancy could not disclose. Those who chose to disclose felt that the partner ought to know their HIV test results. For these women, disclosure to their partners was the most appropriate thing to do albeit for different reasons. However, for most of these women, the acceptance of their HIV status depended on the relationship with the father of the baby. They wanted to live freely without hiding their status from their partners.

It's a huge thing to discover that one has HIV. So, the partner needs to know everything about my life so that in the future, if I do…, or something happens…, he knows everything. I told him the very same day I learned about my status. (Thandy, 22 years old)

I told him after I left the clinic, after I realized that I am pregnant and have to attend antenatal clinic and the HIV clinic as well. (Dintle, 19 years old)

I disclosed after a month…, we could not meet; we only communicated through the phone. I did not want to share such information over the phone. (Dineo, 19 years old)

#### 3.2.3. Knowing the Partner's Status Facilitates Disclosure

For most of the AGYW, the decision to disclose came easily. Their narratives revealed that knowing the partners' HIV status facilitated positive disclosure experiences for them. Disclosure was easy for those whose partners were HIV positive and were receiving ART medication at the time of disclosure. The AGYW were somewhat certain what their partners' reactions would be since they had accepted their partner's HIV diagnosis when the partner initially disclosed.

I think it is because I was free and just disclosed the same way he was open and disclosed his status to me; he did not keep it a secret. He told me the truth, so I did the same thing and I did not have any challenges at all. (Dudu, 20 years old)

It was easy for me to disclose to him because many a times we would talk about HIV. As I mentioned, he goes for HIV testing time and again. I always say to him jokingly…, you go for the test and when you are HIV positive, I will just go and queue for ARTs. So, when I finally found that I am positive, it was not difficult to tell him. (Nthateng, 18 years old)

In contrast, disclosure was difficult for AGYW who did not know the HIV status of their partners. For them, disclosure was difficult since the partner's reaction to disclosure was uncertain or was expected to be negative.

It was difficult, I did not even know where to start to tell him, I told my sister first and she was the one who advised me on what to say. (Lovely, 19 years old)

It was difficult but I ended up letting him know. I was afraid to disclose to him but then in the end, I felt I had…, I had to let him know. (Nthateng, 18 years old)

It took me a month to disclose to him, I did not know how to put it. (Sasha, 18 years old)

The fear of negative partner reaction resulted in keeping the HIV test results secret. One woman had this to say:

My view is for him to disclose his status to me first-so that I may know and after I have learned about his status, I might disclose mine to him. (Dudu, 19 years old)

In addition, the five AGYW who were abandoned when they informed the father of the baby about the pregnancy could not disclose. This is what one had to say:

At the moment, I don't think and see the importance of letting him know because we are no more seeing each other since we separated. I don't think we will ever meet again. (Sasha, 18 years old)

#### 3.2.4. The Need to Protect the Partner

The AGYW chose to disclose to the father of the baby so that they would be provoked to test for HIV and take preventive measures against HIV if they tested negative or to prevent reinfections if they tested HIV positive.

I disclosed to my partner because he will not discriminate, belittle, talk about my status to other people, nor divorce me because I have HIV. He may support me at all times and comfort me. I thought that disclosing could make him want to know his status. (Zanele, 19 years old)

I disclosed so that he may know as well and take a step to go and get tested. (Lovely, 19 years old)

Furthermore, the AGYW chose to disclose to protect their partners from HIV infection. They felt that the partners had to know about their HIV status because they were at risk of being infected with HIV.

I felt I needed to let him know to be protected…, he needed to get tested as well and know his status. Again I wanted us to do things differently from how we used to [use condoms] do before for his protection, I felt I needed to let him know. (Nthateng, 18 years old)

I felt it was important for him to know so that he can be tested and if he test HIV negative he might be given PREP treatment to decrease the chances of HIV transmission. (Nthateng, 18 years old)

So that I do not transmit the diseases to him because he has to come here [hospital] for circumcision so that he does not contact HIV like me. (Puleng 19 years old)

#### 3.2.5. Feeling Relieved

Disclosing to the father of the baby allowed the AGYW to unburden themselves. There was a sense of relief and feeling that a heavy burden was removed from their shoulders after disclosure. Disclosing eased the pain of the HIV diagnosis and the reality of living with HIV. They experienced disclosure positively, and the benefits that they derived from disclosure were of great importance in their acceptance of their test results.

*By the time I disclosed my status there was a huge burden on my shoulders and I felt I needed to off load so I was able to finally do it. I feel* (sighs) *I do n't know truly…, but I felt that once I disclosed my status it made me feel free. I mean it made me feel like any other person. (Thandy, 22 years old)*

After disclosing my status, I felt relieved because I had taken out the pain that I was feeling inside. (Dintle, 19 years old)

It makes me feel as though my heart is healing a bit. (Zinthle, 23 years old)

#### 3.2.6. Disclosure to Facilitate Partner Testing

One other positive outcome of disclosure for the AGYW is that most partners reacted positively to the disclosure. As mentioned, disclosure was to provoke the partners to undergo HIV testing, and the AGYW indicated that their partners underwent the test for HIV after disclosure. This resulted in open discussions about HIV and adoption of safe sexual practices.

After I tested HIV positive and disclosed my status to my partner, he told me he also wanted to test, and he said, “We are going to do it together”. We were both tested. (Thandy, 22 years old)

I was afraid to disclose to him but then in the end I felt I had…, I had to let him know, and I told him I am HIV positive. I told him I am HIV positive and that it is best for him to be tested too. So he tested, but he was negative. (Dintle, 19 years old)

#### 3.2.7. Feeling Supported by the Partner

The AGYW who disclosed indicated that they experienced support and encouragement from the father of the baby. Although the reason for disclosure seemed to be for the benefit of the father of the baby, support is an expectation of a positive disclosure outcome. Even though their need for support was not explicit, AGYW seemed to disclose in order to obtain support. The support received from the father of the baby benefited the AGYW and facilitated their acceptance of their HIV positive results, adherence to ART, and antenatal care.

When I found out that I was positive…, HIV positive and he was HIV negative I was so frightened but later I accepted because after disclosure, he gave me support…. He advised me and he also told me about a lot of stuff that I have to take my treatment every day, he would tell me not to even skip a day without taking the treatment, just like that. (Thandy, 22 years old)

Since my status disclosure he even takes initiative to remind me that it is time for treatment refill - it is time to take the treatment and ask how I am holding up, sometimes when I am going to the clinic for refill, he would accompany me. (Dineo, 19 years old)

I disclosed to my partner so that he may support me at all times and comfort me. (Zanele, 19 years old)

## 4. Discussion

The study explored the disclosure experiences of AGYW after receiving a positive HIV test result during pregnancy. The AGYW visited the clinic to confirm pregnancy and to receive antenatal care, but they were also offered HIV testing as a routine cascade of care in the PMTCT programme [32]. They perceived HIV testing in antenatal care as compulsory and that they had no choice in the matter. A Ugandan study reported that AGYW thought that the test was compulsory and that they could not receive any ANC services unless they accepted being tested for HIV [33]. The Uganda study found that the women fully understood the benefits of HIV testing or PMTCT, while the AGYW in this study understood that they can prevent HIV from being transmitted from the mother to the baby by taking treatment.

Overall, at the time of their interviews, ten of the 15 AGYW reported that they disclosed their HIV status in the immediate posttesting period. A good relationship with one's spouse was identified as a facilitator of disclosure of HIV status to the spouse in other studies. Married women or those in stable cohabiting relationships reported disclosure to the father of the baby to a greater extent compared with single women [13, 15, 20, 21, 34]. Furthermore, a few AGYW knew the HIV status of their partners prior to testing HIV positive and, consistent with other studies, knowledge of a partner's HIV status facilitated disclosure [13, 34].

Five AGYW did not disclose due to the permanent separation from their partners after they had informed the partner about the pregnancy. These AGYW indicated that they would not disclose even if they were still in a relationship because they anticipated that their partners would react negatively to the disclosure. This further supports the view that women disclose when they feel safe in the relationship to do so [21, 35]. The literature suggests that the decision to disclose or not might be more related to how secure particular women feel in the relationship than whether or not they were legally married [36–38]. The study found that the pregnancy for the AGYW who were abandoned by their partners was unplanned and the length of the relationship was very short. Knettel et al. [15] reported similar findings that unmarried women who had not disclosed in their study, consistently expressed fears of being abandoned by a partner if they disclose. In a previous study by the lead author, seven women were abandoned by their partners when they informed them about the pregnancy [21]. The termination of relationships during pregnancy speaks to the instability of partnerships in many settings, which makes HIV disclosure decisions challenging [20].

The decision to disclose to the partners was driven by different needs or concerns for the AGYW. One of the main reasons the AGYW chose to disclose was to protect the partners from HIV infection. Implied in the need to protect the partners was the hope that the partner would then be tested for HIV and take preventive measures against HIV if they tested negative. Concerns for the partner's health was the major reason cited for disclosing to sexual partners in other studies [14, 21].

The study found that the AGYW discovered their partner's status upon disclosing their own status, which indicates some level of success in encouraging their partners to test. This was one of their key reasons for disclosing to their partners. Watt et al. [20] reported comparable reasons for disclosure to partners and indicated that women saw HIV testing as a way for the partner to get treatment and initiate conversations around HIV. Following partner testing, the AGYW in the study reported that they adopted safe sex practices to prevent infecting the HIV negative partner or reinfections. Similar safe sex practices were observed in another study conducted with women in a PMTCT programme [39]. The desire to have the partner test for HIV could be a point of intervention for PMTCT services, as disclosure to partners increases women's participation in PMTCT programmes [14].

The AGYW recounted an overall positive experience of disclosure in that the reactions to disclosure by their partners were positive. Consistent with other studies, the AGYW described feeling as though a burden had been lifted and that disclosure brings freedom and relief as they do not have to keep secrets which brings worries and stress [20]. Furthermore, disclosing eased the sorrow and sadness of the HIV diagnosis and the reality of living with HIV. The AGYW in the current study and other studies wanted to live freely to take their ART medication without hiding them from their partners [20]. This was particularly important for those in marital relationships who wanted to avoid the stress of keeping a secret or hiding their ART medication from their spouses.

The outcome of disclosure for those who disclosed to their partners was support to adhere to ART medication and clinic attendance. The AGYM mentioned support in the form of reminders to take their ART, providing transport money for clinic attendance, accompaniment to the clinic for antenatal care, and emotional support to deal with the different phases of being HIV positive and pregnant. Other studies in SSA reported similar findings that partners are generally supportive after disclosure [13, 15, 20, 40].

### 4.1. Study Limitations

As with all qualitative studies, the study sample was a small number of participants; therefore, the findings cannot be generalised to the whole country. Furthermore, the study included AGYW from one district in the country and their experiences may or may not be similar to other AGYW in other settings. However, the study findings provide an understanding of the outcomes of disclosure in pregnancy. The study cannot rule out social desirability in reporting the outcome of disclosure to partners since none of the AGYW reported negative partner reaction. The study focused on disclosure to partners and falls short on reporting incidences of stigma that are associated with nondisclosure in quantitative studies in SSA.

## 5. Conclusions

The study has provided a description of the disclosure experiences of AGYW who test positive and disclose in pregnancy. Although not all the AGYW in the study disclosed to the father of the baby, those who did had positive experiences of disclosure. The outcome of disclosure for most was support by the partner to adhere to ART medication and other PMTCT interventions. The AGYW were supported despite some of their partners having tested negative. The fact that all the partners agreed to test for HIV and adopted safe sex practices has implications for the success of the PMTCT programmes.

There is a need for interventions to enhance disclosure and for partner testing to be integrated into the cascade of services in PMTCT programmes. This is of particular significance since the counselling provided within the PMTCT programmes does not address the disclosure challenge that AGYW face.

## Figures and Tables

**Table 1:** Sociodemographics of adolescent girls and young women.

Variable	Subcategory	Frequency
Level of education	Primary	3
	Secondary	10
	Tertiary	2
Marital status	Single	5
	Married	10
Family structure	Living with parents	5
	Living with partner	9
	Living with in laws	1
Number of pregnancies	One	14
	Two	1
Employment status	Employed	0
	Unemployed	15
Disclosed to partner	Yes	10
	No	5
Disclosed to family members	Yes	15
Partner's status	Positive	5
	Negative	5
	Unknown	5
Relation status	Ongoing	10
	Terminated	5

## Data Availability

The data used to support the findings of this study are included within the article.
